# Decreasing the duration of untreated illness for individuals with anorexia nervosa: study protocol of the evaluation of a systemic public health intervention at community level

**DOI:** 10.1186/s12888-014-0300-1

**Published:** 2014-11-18

**Authors:** Antje Gumz, Natalie Uhlenbusch, Angelika Weigel, Karl Wegscheider, Georg Romer, Bernd Löwe

**Affiliations:** Department of Psychosomatic Medicine and Psychotherapy, University Medical Center, Hamburg-Eppendorf & Schön Klinik Hamburg Eilbek, Martinistraße 52, 20246 Hamburg, Germany; Department of Medical Biometry and Epidemiology, University Medical Center Hamburg-Eppendorf, Martinistraße 52, 20246 Hamburg, Germany; Department of Children and Adolescent Psychiatry, Psychosomatics and Psychotherapy, University Medical Center Münster, Germany, Schmeddingstr 50, Münster, 48149 Germany

**Keywords:** Anorexia nervosa, Duration of Untreated Illness, Treatment Initiation, Systemic public health intervention

## Abstract

**Background:**

Anorexia nervosa (AN) is a mental disorder with grave burdens for affected individuals as well as for the healthcare system. One of the strongest predictors of a poor outcome is a long Duration of Untreated Illness (DUI), which is defined as the time between the onset of the disease and treatment initiation. Reducing the DUI is an important step to optimize care of individuals with AN. In order to achieve this aim, systemic public health interventions are necessary. Objective of this study is to evaluate a systemic public health intervention at Community level aiming to reduce the DUI in individuals with AN.

**Methods/design:**

The intervention includes the establishment of a network of health care professionals within the area of eating disorders (EDs), the development of an internet-based treatment guide, the presentation of informative short-films about EDs in cinemas and a corresponding poster campaign as well as a special outpatient clinic. For the evaluating study a pre-post between-subject design is chosen. The DUI, and the duration until first contact (DUC) with a health care professional, ED pathology as well as comorbidity are assessed before and after the systemic intervention is carried out.

**Discussion:**

The study attempts to provide evidence of the effectiveness of an ED-related systematic public health intervention. Additionally, the study will lead to a better understanding of the DUI, which is essential in order to improve care of individuals with AN.

**Trial registration:**

Current Controlled Trials ISRCTN44979231; Registered 11 November 2011.

## Background

Anorexia Nervosa (AN) is an eating disorder (ED) mainly characterized by a refusal to maintain body weight, an intense fear of weight gain as well as a distortion of body perception. It can have serious adverse consequences on physical as well as psychological health. Common concomitant effects include cardiac arrhythmias, electrolyte imbalances, renal insufficiency, osteoporosis as well as severe infections [1]. In addition, many patients with AN suffer from comorbid psychiatric disorders (e.g. [2-4]) with anxiety and affective disorders being most frequent [5,6].

Compared with other mental disorders, AN occurs relatively seldom with an average prevalence estimated at 0.3% [7]. However, with 0 to 22% the morbidity and mortality rates are among the highest of any mental disease [8,9]. In a 21-year follow-up study 14% of the patients died from reasons directly related to AN [10]. Moreover, in spite of low prevalence rates, AN generates high costs for the health care systems - almost equivalent to those reported for schizophrenia [11].

Due to the various adverse consequences for AN-affected individuals and the healthcare system an early and effective treatment is indispensable. There is evidence for the effectiveness of treatment methods for AN [12]. Nevertheless, estimates show that only half of the AN cases are detected in primary care settings [13] and only one third of the cases detected receive treatment [7]. There can be different reasons for AN- patients to remain untreated. Practitioners might not discover the disorder or patients might decide against a recommended treatment due to the ego-syntonic nature of AN [14].

Even after a successful treatment initiation, there are several factors that may have a negative impact on the course of the disease. The most important predictors of a poor outcome are a low Body-Mass-Index (BMI) as well as severe psychological and social problems [10]. Some studies report that a higher BMI at treatment initiation is the strongest predictor of a successful course [15,16]. Therefore, treatment needs to be initiated before weight loss becomes too severe and protracted [17]. This emphasizes another relevant outcome predictor: the Duration of Untreated Illness (DUI). The DUI describes the time between the onset of an illness and the initiation of treatment. There is evidence for a modest association between DUI and outcome in schizophrenia [18,19] and some indication of an association in mood and anxiety disorders [20,21]. The DUI may also play an important role in AN- patients (e.g. [22,23]). It can be concluded that treatment is more likely to be successful when patients have suffered from AN for a shorter period of time [24]. A review containing six studies from different Western countries found the average DUI to be 1,78 years in patients with AN [25]. This is of great clinical concern as a long duration of AN may be associated with severe complications such as increased mortality [26].

So far, little research has been conducted on factors predicting the DUI in AN. Ackard and colleagues [27] reported the duration of illness to be longer in middle-aged (≥40 years) than in younger women (18–39 years). Healthcare system related aspects influencing the time between symptom onset and treatment utilization are sufficient information about diagnostic and treatment options, availability as well as networking of different care givers [28,29]. Especially general practitioners and pediatricians need to have competence regarding diagnostic of and treatment for EDs to facilitate and accelerate referral into specialist care [30].

There are several interventions and resources available to effectively treat AN [12] which are currently not used to the full extent [7,13]. The reasons for the existing potentials not being exploited fully may be found in individuals as well as systemic structures. Thus, interventions aiming to take this complexity into account have to be multi-faceted as well. In the area of depressions, it has been shown that systemic interventions are able to positively intervene in existing structures. With its four-level intervention program for improving care of patients with depression the “Regensburg Alliance against Depression” succeeded in significantly decreasing suicide rates in Regensburg [31].

### Objectives

The main objective of this project is to minimize the DUI in individuals with AN. In order to achieve the aim of the project, a systemic public health intervention is carried out. It is hypothesized that this intervention decreases the DUI in individuals with AN.

This study is part of the project *psychenet*: *Hamburg network for mental health* funded by the Federal Ministry of Education and Research (BMBF). The purpose of *psychenet* is to work on establishing innovative care models, which aim to make decisive improvements to the prevention, diagnosis and treatment of people with mental illnesses in the Hamburg metropolitan region [32]. *Psychenet* is coordinated by the Cluster Agency Healthcare Hamburg (www.GWHH.de) in collaboration with the University Medical Centre Hamburg-Eppendorf. It consists of a total of eleven sub-projects. Since the network was initiated in 2011, more than 80 scientific and medical institutions, counselling centers, the Senate and the Chamber of Commerce of the Free and Hanseatic City of Hamburg, companies, as well as patients’ and relatives’ associations have been acquired to participate. Sub-project IX (*Health network anorexia and bulimia*) focuses on the improvement of the effectiveness and efficiency of ED-specific care. It aims to optimize the use of available resources as well as the connection of existing structures. The sub-project is divided into two different intervention arms. This article will provide an overview about one intervention arm focusing on treatment initiation in AN patients. An overview about the second intervention arm (a randomized controlled trial on the prevention of EDs in schools) will be described elsewhere.

## Methods

### Study design

**Figure 1:**
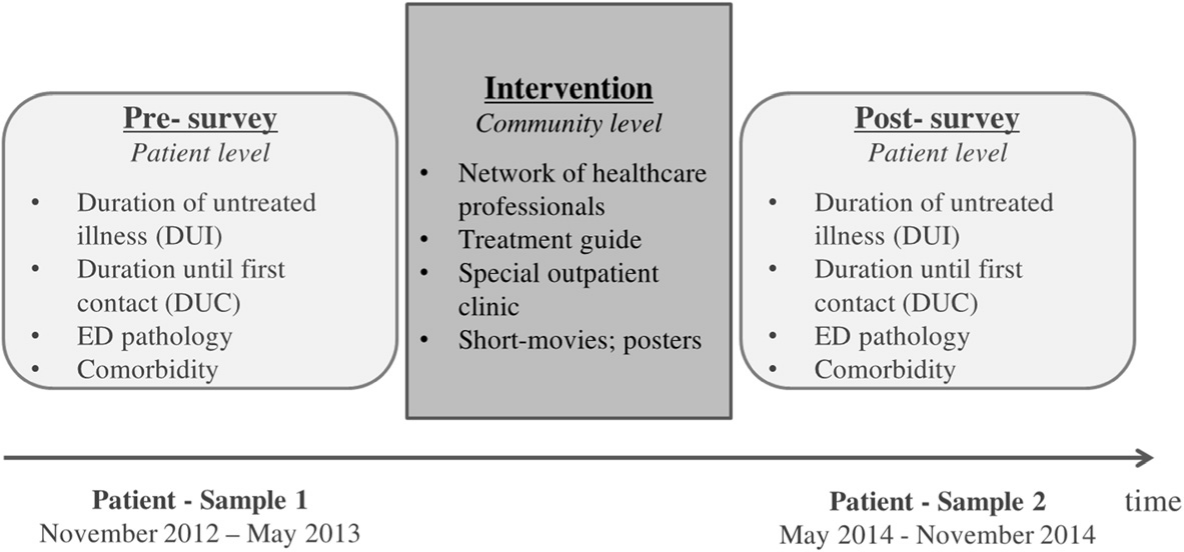
**Course of data collection.** Note. Recruitment for pre- and post-survey occurred within the same institutions offering evidence-based treatment for AN based on the German S3-Guidelines for EDs [35].

For the study purpose, a pre-post between-subject design was chosen. DUI, duration until first contact (DUC), current ED pathology and comorbidity are measured before and after the implementation of the systemic intervention. The pre-data collection takes place between November 2012 and May 2013. Twelve months are planned for the systemic intervention. The post-data collection takes place between May 2014 and November 2014. Figure 1 illustrates the course of the data collection.

### Ethics approval

The study procedure was reviewed and approved by the ethics committee of the Psychotherapist Chamber of Hamburg.

### Study procedures

#### Inclusion and exclusion criteria

In order to participate in the study, a signed informed consent is necessary. Patients below 16 years are required to provide additional informed consent from a parent or legal guardian. Moreover, participants need to have female sex, should be a minimum of ten years old and have a treatment diagnosis of typical or atypical (one criterion absent) AN according to the DSM-IV [33]. Furthermore participants should currently receive their first ED-specific in- or outpatient treatment or be current client of an ED-specific counselling center. Exclusion criteria are insufficient knowledge of the German language as well as substantial organic or psychological complaints preventing participation from the recruiting practitioner’s point of view.

#### Recruitment

The recruitment is coordinated in cooperation with different ED-specific treatment settings in Hamburg and adjacent areas, i.e. inpatient wards, day clinics, outpatient departments and ED-specific counselling centers. Included institutions offer evidence-based treatment for AN based on the German S3-Guidelines for EDs [34]. A total of eleven clinics, nineteen outpatient departments and four counselling centers are asked to support the project with recruiting participants. A special network meeting is used to inform cooperating practitioners about the study’s content, procedure, and recruitment as well as to provide practitioners with study materials (e.g. written information about the course of recruitment, study design, inclusion and exclusion criteria as well as informed consent forms and questionnaires for participants). During the study, cooperating practitioners regularly receive phone calls to settle any questions and promote adherence.

Cooperating practitioners select eligible participants in line with inclusion and exclusion criteria. Participants found eligible are verbally informed about the aims and procedure of the study and are additionally provided with written material. In case of willingness to participate, participants are asked to sign the informed consent. Practitioners then hand out the questionnaire set, which is described later in this paper. All cooperating practitioners are required to return the completed questionnaires as well as the signed informed consents to the research team. Once all documents are complete, participants are contacted for a structured telephone interview.

The procedure will be identical in the post-survey. Participants who participated in the pre-survey are excluded from participation in the post-survey. Between the two points of data collection the systemic intervention takes place.

#### Modules of the intervention

Between 2011 and 2014 the research network *psychenet*: *Hamburg Network for Mental Health* implements different illness specific and general population oriented interventions and evaluates the resulting systemic interventions. The *psychenet* sub-project “*Healthcare Network Anorexia and Bulimia nervosa*” establishes a systemic ED-specific intervention containing the following components:

- The establishment of a network of health care professionals within the area of EDs
- The development of an internet-based treatment guide for EDs
- The presentation of informative short-films about EDs in several cinemas as well as a corresponding poster campaign
- A special outpatient clinic

The multidisciplinary network connects different healthcare professionals within the area of EDs. Practitioners from different settings with a medical and/or a background in clinical psychology regularly meet to exchange scientific knowledge as well as clinical experiences concerning AN treatment. The internet-based treatment guide (www.essstoerungen.psychenet.de) comprises information about EDs in general as well as treatment options and contact information of ED-specialized in- and outpatient institutions. The recommendations are based on the German S3-Guidelines for EDs [34]. The guide addresses individuals suffering from AN, relatives and healthcare professionals. In order to reach patients with a migration background as well, it is translated into English and Turkish. Components of the systemic intervention targeting the general population are the presentation of a short-film (see www.youtube.com/watch?v=6VPvCp_WWvI&index=2&list=PLF8DFEFE29B837EFC) in cinemas as well as a corresponding poster campaign. The poster-campaign comprises citations of the principal performer of the short-film with an eye-portrait and a short note about the prevalence of mental illness as well as a reference towards the main homepage (www.psychenet.de).

### Variables and instruments

The data is collected in a two-stage course of action with an antecedent questionnaire followed by a telephone interview. The questionnaire contains standardized instruments to measure ED pathology and comorbidity, as well as a set of questions concerning sociodemographic information, disorder onset and the initiation of treatment. The telephone consultation is started off with a diagnostic interview reviewing AN diagnosis by using the Structured Clinical Interview for DSM-IV SCID [35]. Then, questions regarding the onset of the disorder and the initiation of treatment follow to verify information collected from the questionnaire. The onset of the disorder is confirmed by requesting information about the beginning of weight loss, beginning of fear to gain weight, beginning of distortion of the body perception and the initial absence of the menstruation. In an exploratory part reasons for the individual DUI are explored.

#### Duration of untreated illness (DUI)

The DUI is understood as the number of months between illness onset and the initiation of an ED-specific treatment. Onset of the disease is conceptualized as the first occurrence of all symptoms of typical or atypical AN as described above.

#### Duration until first contact with healthcare system (DUC)

The duration until first contact (DUC) is conceptualized as the number of months between illness onset and the first contact with the healthcare system due to ED-related issues initiated either by the patient or the practitioner. Before starting an ED-specific treatment, patients often have several initial contacts with healthcare professionals or advice centers. The variable DUI can not take these contacts into account because it is defined as the duration until the initiation of a guideline-based treatment. The DUC is meant to improve the understanding of the processes preceding treatment initiation. The questionnaire lists different potential therapeutic settings for AN (inpatient, day-clinic, outpatient, ED-specific counselling) and assesses which of these have been consulted to get information and/or support concerning AN and, in each case, when the first contact took place.

#### ED pathology

ED pathology is measured by the BMI in combination with the German version of the *Eating Disorder Examination Questionnaire EDE*-*Q* [36]. The BMI is calculated from body weight and height. Current weight is requested in the questionnaire as well as in the telephone interview. Additionally, weights at disorder onset, at treatment initiation as well as at point of lowest weight are assessed in the telephone interview. Given the community-based character of the present study, it is not feasible to get externally validated weight measurements for all participants. Additionally, since patients with AN in in- and outpatient treatment institutions are weighted weekly, self-reported weight will be considered adequate. The *EDE*-*Q* assesses specific ED psychopathology on four subscales, which are restraint, eating concern, weight concern, and shape concern. For patients below the age of sixteen, the child version *Ch*-*EDE*-*Q*, [37,38] is used. The psychometric criteria of the *EDE*-*Q* are satisfactory. Objectivity is given and the test is very reliable with an intern consistency of α = .97 and a retest-reliability of r = .88. Validity could be confirmed as well [36].

#### Comorbidity

Comorbid psychopathology is assessed by the depression module of the *Patient Health Questionnaire* (*PHQ*-*9*, [39-41]) and the *Generalized Anxiety Disorder Scale* (*GAD*-*7*, [42]). The *PHQ*-*9* is a self-assessment scale consisting of nine items. It is able to establish provisional depressive disorder diagnoses and grade depressive symptom severity [43]. There is strong evidence for the reliability of the instrument with an intern consistency of α = .89 and a retest-reliability of r = .84 [44]. The *GAD*-*7* is a self-assessment anxiety scale consisting of seven items. It has good reliability as well as criterion, construct, factorial, and procedural validity [42,45].

#### Control variables

The questionnaire includes sociodemographic and control variables, such as age, gender, marital status, educational level, migration background, housing situation and occupational activity. At post assessment, patients are asked to indicate any contact with parts of the systemic interventions from *psychenet*. In case of positive response, further questions clarify which of the ED-specific interventions were recognized and if treatment initiation is subjectively eased by these interventions.

### Sample size/power calculation

Based on former international studies [25] with an average DUI of 19 months and an effect size of 0.4, we considered a DUI reduction of 4.8 months or 25% as clinically relevant. Thus, for a two-sided type-I error of 5% two samples with 100 participants each are needed to reach statistically significance with a power of 80%.

### Primary outcome

The primary outcome is the DUI, defined as the time in months between the onset of AN and first specific treatment.

### Secondary outcome

As secondary outcome, DUC, current ED pathology as well as comorbid depressive or anxious psychopathology are assessed. Depression and anxiety are the most frequent comorbid psychiatric disorders accompanying AN (e.g. [5,6]).

### Study hypotheses

It is hypothesized that the systemic intervention of *psychenet* decreases the DUI in the post-group compared to the pre-group. As described above it is of great relevance to detect and treat individuals with AN sooner. Next to individual factors such as age [27] there are systemic aspects like sufficient information, availability and networking of different components of the health care system influencing patient flow into ED-specific care. The intervention focuses on spreading information about EDs, informing about paths to treatment, improving the networking of different healthcare professionals and extending the offer by establishing a special outpatient clinic. Therefore, it seems reasonable to assume that the systemic intervention decreases the duration until an ED-specific treatment is initiated. For the same reasons it is hypothesized that the systemic intervention decreases the DUC. Moreover, it is assumed that the systemic intervention leads to a reduced ED pathology as well as a reduced comorbidity with depression and anxiety at the beginning of treatment. It is further suspected that this effect is mediated by the DUI. An earlier intervention and a shorter duration of illness prior to treatment initiation may lead to a reduced severity of AN and a reduced comorbidity because treatment starts before a chronification can occur. Therefore, it is conceivable that the expected shorter DUI in the post-group leads to a decreased ED pathology and comorbidity at treatment initiation. Due to the lack of previous research these hypotheses will be analyzed exploratorily.

To display them at a glance, the hypotheses of the study are:

- The systemic intervention decreases the DUI in the post-group compared to the pre-group.
- The systemic intervention decreases the DUC in the post-group compared to the pre-group.
- The systemic intervention decreases the ED pathology at treatment initiation in the post-group compared to the pre-group via DUI.
- The systemic intervention decreases the comorbidity with depression and anxiety at treatment initiation in the post-group compared to the pre-group via DUI.

**Figure 2:**
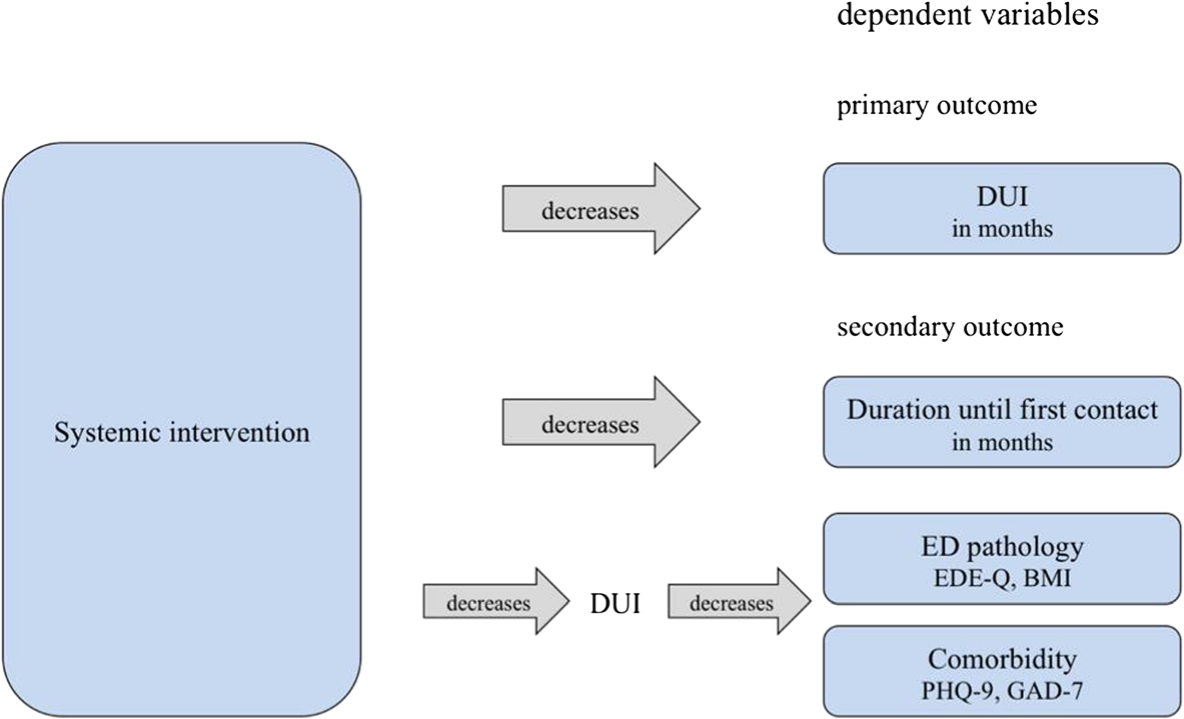
**Expected impact of the systemic intervention on the primary and secondary outcome parameters.**

Figure 2 illustrates the expected impact of the systemic intervention on the primary and secondary outcome parameters.

### Handling of missing values

Participants who appear unavailable for the telephone interview will be regarded as withdrawal. Analyses will be performed on completers due to the exploratory approach of the study. Additionally, sensitivity analyses using imputed data (fully conditional specification [46]) will be conducted.

### Statistical analyses

In order to test the primary hypotheses regarding change vs. no change from pre to post assessment univariate multiple regression analysis with the dependent variables DUI and DUC as well as the independent variable period of assessment will be conducted. Additionally, recruitment setting will be included as random control variable whereas age and contact with the systematic intervention will be taken into account as a fixed control variable. Statistical significance will be taken two-sided p <0.05 throughout and regression coefficient with 95% confidence intervals (CI) will be used to express the uncertainty in the data.

## Discussion

This study is a pre-post study that examines the evaluation of a systemic public health intervention for EDs. The systemic intervention contains the establishment of a network of health care professionals within the area of EDs, the publication of a treatment guide for patients, relatives and therapists, the distribution of information concerning EDs by short-films and posters as well as the establishment of a special outpatient clinic. It is expected that the systemic intervention decreases the time between the onset of AN and the first contact with healthcare system as well as the beginning of an ED-specific treatment. It is further assumed that the systemic intervention reduces ED pathology and comorbidity at treatment initiation. Currently, there is no comparable intervention in the field of EDs.

The value of the intervention must be classified as high since a long DUI in AN is one of the strongest predictors of a poor outcome [16]. Evidence-based interventions (e.g. [47]) have to be initiated at an early stage of the illness to develop the full effectiveness. Reducing the DUI is one important step to improve patient flow into special health care in patients with AN. Moreover, the intervention plans to improve the networking of different instances of the health care system. Use of existing resources is increased, which could on a long-term basis lead to a reduction of the high costs for AN treatment [11]. At present, evidence-based treatments for AN [12] are not utilized sufficiently early and targeted [7,13,25,48].

The study aims to evaluate the systemic intervention, which is, next to the value of the intervention itself, of great interest for further public health interventions. Assessing the DUI and additionally the DUC may also lead to a better understanding of the period during the time until the first ED-specific treatment is initiated.

In spite of the huge demand for such evaluated interventions, some limitations to this study have to be mentioned: Since this project is a systemic public health intervention, the implementation of a Randomized Controlled Trial (RCT) is not feasible. Therefore, it is problematic to control confounding factors and make causal conclusions. However, this limitation can be attributed to the aims of the project, which leaves no option of implementing a RCT. Due to the complexity of the systemic intervention it is further not realizable to conduct a dismantling investigation. It is assumed that some parts of the intervention act unconsciously. Therefore an entire understanding of the underlying dependencies will not be possible. Moreover, it is decided to exclude male patients from participation - admittedly at the expense of generalization of the results. The reasons for this decision can be found in the low prevalence of AN in males. It has to be noted that there is a lack of standardized measures assessing DUI and DUC. This makes it necessary to develop a new measure for the purposes of the study. Additionally, the variables of interest mainly can only be assessed retrospectively. To counteract a possible distortion, self-reported data is reviewed in the telephone interview. In addition, it has to be considered that a prospective study would not be feasible due to the low prevalence of AN. There are some further limitations concerning the recruitment of the participants. There may be an adherence bias of the cooperating practitioners leading to different participation rates. There may be also a selection bias: Chronified patients with extremely low body weight may display a very long DUI but may be excluded due to their current physical status. The consequence may be an underestimation of the average DUI in the sample.

Since there hasn’t been a comparable intervention in the field of AN, some hypotheses have to stay exploratory. However, the effectiveness of public health interventions in general could be shown already (e.g. [31]). In the field of EDs the *psychenet* intervention and its evaluation will gain new knowledge about the DUI and factors influencing this period. Also, the successful implementation of a systemic intervention in this area will improve the networking of different health care professionals and facilitate patients’ flow into specialist care.

## Abbreviations

AN: Anorexia nervosa
BMI: Body-mass-index
CI: Confidence intervals
DUC: Duration until first contact with healthcare system
DUI: Duration of untreated illness
EDs: Eating disorders
BMBF: Federal Ministry of Education and Research
